# The influence of organizational factors on the attitudes of residential care staff toward the sexuality of residents with dementia

**DOI:** 10.1186/s12877-018-1023-9

**Published:** 2019-01-08

**Authors:** Tineke S. M. Roelofs, Katrien G. Luijkx, Marielle C. M. Cloin, Petri J. C. M. Embregts

**Affiliations:** School of Social and Behavioral Sciences, Department of Tranzo, Tilburg University, Schakelring, Waalwijk, Tilburg, the Netherlands

**Keywords:** Dementia, Sexuality, Care staff attitudes

## Abstract

**Background:**

The attitudes of care staff toward the sexuality of residents with dementia they care for is assumed to influence the residents’ expression of their sexuality in the way they want. This paper examines the effect of organizational factors, person-centered care, and the culture of the organization on the attitudes of care staff toward the sexuality of residents with dementia in residential care facilities (RCF) .

**Methods:**

Care staff in different functions at six RCF organizations (*N* = 187) participated. Using a survey, we gathered information on demographics and care-staff careers, attitudes toward resident sexuality, the culture of the organization, person-centered care, and knowledge of resident sexuality. Ordinary least square (OLS) hierarchical analyses were performed to analyze results.

**Results:**

Care staff attitudes were found to be positively affected by person-centered care, and marginally positively affected by a supportive culture in the organization, Moreover, knowledge of resident sexuality positively affected care staff ‘attitudes toward resident sexuality, and the presence of policy regarding resident sexuality affected them negatively .

**Conclusions:**

Despite different study limitations, these results give a first insight in a broad perspective on care staff attitudes toward resident sexuality. In addition to improving knowledge of the care staff, enhancing person-centered care and a supportive culture in the organization will improve care-staff attitudes toward resident sexuality.

## Background

Positive intimate and sexual experiences are found to influence health and quality of life (QoL) positively in the elderly [1–3]. However, for people with dementia, enhancement of these positive experiences is not straightforward, especially not for those living in residential care facilities (RCF). Because residents with dementia depend greatly on the care staff in many areas, including sexuality [4], attitudes of the care staff are expected to influence if and how residents are able to express their sexuality [5]. As such, attitudes of the care staff might also be a barrier to sexual expression of residents with dementia [6].

A small body of research exists on caregiver attitudes toward the sexuality of residents with dementia, based on the assumption that a more open or positive attitude positively influences the expression of sexuality by residents [7]. Although general neutral or positive attitudes were found among direct caregivers [8–11], care staff in general also expressed great and diverse concern regarding the sexuality of residents with dementia [9, 12, 13]. These concerns caused feelings of discomfort, which in turn might lead to the denial of residents’ sexual needs or labeling sexual behavior as problematic [4, 7, 13–15].

Several factors at the level of the individual caregiver and their careers were reported to influence their attitudes toward resident sexuality. First, age was an influence. Older employees had more positive attitudes toward sexuality of residents with dementia than their younger colleagues [16]. This difference was attributed to the smaller age gap between the residents and older caregivers. Second, a higher level of education [10, 16] and less religious adherence [10] were found to enhance a more positive attitude. Finally, more knowledge of the sexuality of residents with dementia was associated with a more positive attitude of caregivers [10]. In another study, a training program on intimacy and sexuality in residents was found to increase this knowledge [17]. However, it is unknown if more knowledge leads to a more positive attitude toward resident sexuality, or vice versa. Possibly care staff with positive attitudes are also more willing to increase their knowledge of this topic. Throughout the literature, the absence of training programs was highlighted [18], and the importance of improving attitudes and reducing stigma directly in training programs, rather than just providing more knowledge, was emphasized [10].

In addition to these individual factors and career characteristics (e.g. participation in a training program), previous literature has emphasized the importance of factors at the level of the organization [9, 13, 14]. Based on a qualitative study, Roach (2004) concluded that attitudes toward resident sexuality are part of a broader perspective labeled as the “Guarding Discomfort Paradigm” [13, 14]. This paradigm implies that care staff, reactively or proactively, try to avoid or decrease their own feelings of discomfort about resident sexuality. A supportive culture in the organization was proposed to have a positive influence on these feelings of discomfort and consequently on care staff attitudes toward resident sexuality. This culture of the organization can be defined as the organization’s character and norms, based on a wide range of social phenomena [19]. Next, the importance of the presence of policy and guidelines with regard to resident sexuality has been emphasized throughout the literature, because they form part of the culture of the organization and reflect the importance a care organization attributes to resident sexuality [9, 18, 20].

In addition to the organizational culture, in recent years the concept of person-centered care has generally gained popularity in dementia care. In this concept, care is meant to be holistic and empowering, and should aim at increasing resident QoL [21, 22]. This development requires organizations and care staff to consider the sexual needs of residents as a part of their basic human needs. Consequently, an increase in the provision of person-centered care might lead to more positive attitudes of care staff toward resident sexuality. To our knowledge, no research has examined this assumption. Moreover, in the literature on person-centered-care, sexual needs have not yet been considered.

The aim of this study is to examine in a broad perspective the attitudes of care staff toward the sexuality of residents with dementia, by examining the possible influence of organizational factors on care staff attitudes. In this study, organizational factors are operationalized as person-centered care and the culture of the organization. Next to the organizational factors, the role of individual factors, knowledge of resident sexuality, and some characteristics of care staff career (e.g. years of tenure and current function) over the possible effect on the main target variable “attitude” are included.

## Design and methods

### Setting and participants

The data for this study were collected at psychogeriatric care units of RCFs located in the south of the Netherlands. The RCFs participated in an academic collaborative network with the aim of connecting research and practice to elderly care. The participating RCFs varied in size between 590 and 1000 residents. An admission into a RCF becomes inevitable when the cognitive and physical impairments of patients with dementia rise to severity levels that make care at home with help of a private and formal care network impossible. In the Netherlands, this highly intensive care is provided in psychogeriatric care units. These are protected living environments, where approximately six to ten persons with moderate to severe dementia reside in a closed unit.

Employees with both direct and indirect contact with residents of such psychogeriatric care units were recruited. Direct caregivers are employees with a vocational level of education, who work together in a care team that belongs to a specific psychogeriatric unit and so provide direct daily care to residents with dementia. However, Dutch psychogeriatric care is organized in a multidisciplinary way. This means that several professionals with different expertise and tasks, and mostly higher levels of education, are indirectly involved in the care of residents (e.g. managers, therapists, such as physiotherapists, medical doctors, and psychologists). Although RCF care staff other than direct caregivers are less involved in daily care, they are mostly responsible for policy, guidelines, and treatment or care advice for the direct caregivers.

Initially, a convenience sample of 191 employees participated in the study. One minor (< 18 years of age) was excluded because of considerations concerning the ethical review. Two respondents were excluded because they barely filled in the questionnaire (< 10% of the questionnaire). To maximize the sample size in analyses, we included all cases for which 80% of the items on the dependent variable, attitude toward resident sexuality, were completed. This resulted in a final sample size of 187.

The participant characteristics are shown in Table 1. The sample was mostly female (*n* = 179, 95.7%). Mean age was 40.8 years (range 18–64), and they had an average of 16 years of tenure within care (range 1–43). Most of the participants completed an average vocational education and worked as direct caregivers.

**Table 1:** Participant characteristics

		N (%)	M (SD)
Age (*n* = 186)^a^			40.8 (13.1)
Years of tenure *(n* = 185)^a^			16 (11.6)
Level of education^a^			
	Low vocational	27 (14.7)	
	Average vocational	83 (43.5)	
	High vocational	33 (17.3)	
	High	41 (18.4)	
Current function			
	Caregiver	135 (72.2)	
	Therapist	24 (12.8)	
	Other	28 (14.9)	
Employer (organization)^a^			
	A	28 (15.7)	
	B	40 (21.5)	
	C	19 (9.9)	
	D	44 (23)	
	E	39 (20.4)	
	F	15 (7.9)	
Sexual education^a^	No	155 (6.5)	
	Yes, 1	18 (9.8)	
	Yes, > 1	13 (83.7)	
Policy^a^			
	Yes	75 (44.9)	
	No	92 (55.1)	

^a^*n* does not add up to 187 in all variables because of missing data

### Procedure and measures

The Tilburg University psychological ethics committee granted ethical approval (Reg. No. EC 2015.60), and approval of the executive and ethical boards of the participating organizations was also obtained.

Hard-copy surveys were distributed after scheduled (team) meetings. A convenience sample was gathered through the selection of the meetings, in which all employers were represented. Approximately ten members of care staff attended these meetings and care staff with different functions were present during most meetings. Participation was voluntary and participants could withdraw from the study at any time. The participants received an information letter, an informed-consent form, and the survey for immediate completion. The informed consent form and anonymous questionnaires were stored separately. One author (TR) or a student assistant was present during data collection to answer all questions. The author and assistant aligned their answers in frequent discussions about the data-collection process.

The survey questionnaire was divided into five sections; one includes the main outcome measure (care staff attitudes toward resident sexuality), and four include the covariates. To estimate reliability of the measures based on the sample, we used Cronbach’s alpha test for internal consistency. Values between .7 and .9 were considered acceptable [23].

#### Main outcome measure: Care staff attitudes toward sexuality

The Dutch version of the aging sexual knowledge and attitudes scale (AKSAS) [24, 25] was used to assess care staff attitudes and knowledge of resident sexuality. The attitudes subsection was used as the main outcome measure; the knowledge subsection was used as a controlling variable (see below for details).

The attitude subsection consists of 25 items, rated in a seven-point Likert scale from 1 (totally disagree) to 7 (totally agree), it included such items as “Aged people have little interest in sexuality.” Ten items were reversed after completion of data collection. An example of such an item is “Masturbation is an acceptable sexual activity for older males.” Total scores were calculated ranging from 25 (most positive attitude) to 175 (least positive attitude) for analysis, as used before [24].

For this subsection, the developers of the scale found high internal consistency and reliability (Cronbach’s α = .88) and sufficient content validity (scale-level content validity Kappa = .91), based on the judgment of ten experts [24]. Reliability was sufficient for our sample as well (Cronbach’s α = .83).

#### Main independent variables: Person-centered care, and culture of the organization

##### Person-centered care

Person-centeredness is defined as a holistic view of residents, and in this maintaining personhood, despite increasing cognitive and physical impairments [21]. The person-centered care assessment tool (P-CAT; [26] was used for this study to assess the degree to which employees feel they provide person-centered-care (own assessment). The questionnaire was translated from English to Dutch after a forward and backward translation process [27]. The questionnaire consists of 13 items, rated in a five-point Likert scale ranging from 1 (disagree completely) to 5 (agree completely). Employees were asked to indicate to what extent they think these statements correspond to their own current work experiences. An example of such a statement is “Assessment of residents’ needs is undertaken on a daily basis.” Five items, which were formulated negatively, were reversed after completion of the data collection. Total scores were calculated ranging from 13 (low person-centeredness) to 65 (highest person-centeredness). Internal consistency was assessed to be good in the initial study (Cronbach’s α = .84). Both construct and content validity were also demonstrated to be good [26]. Internal consistency was also sufficient for our sample (Cronbach’s α = .79).

##### Culture of the organization

The FOCUS Questionnaire [28] was used to assess the culture of the RCF organization. This questionnaire was developed based on the competing values model [29] and includes four cultural orientations: support, innovation, rules, and goal orientation. Different aspects of the culture of the organization define the four different orientations. The support orientation is characterized by cooperation, team spirit, and individual growth and is person based. The innovation orientation includes aspects such as creativity, anticipation, experimentation, and searching for new information. The rule orientation includes aspects such as respect for authority and division of work. It also emphasizes a hierarchical structure and communication. Finally, the goal orientation includes rationality, accomplishment, and accountability.

The four cultural orientations are measured from a descriptive and an evaluative perspective. The complete questionnaire consists of 54 items that are distributed over eight different variables (four orientations measured from two perspectives). The descriptive perspective measures directly observable behavior and consists of 25 items rated in a six-point scale (ranging from “never” to “always”). An example of an item is “How often is constructive criticism accepted?” The evaluative part measures the perception of employees regarding typical characteristics of the organization and consists of 29 items, rated on a six-point scale (ranging from “very” to “not at all”). An example of an item is “How typical is mutual understanding?”

Reliability (internal consistency) was assessed for the eight different variables separately [28]. The internal consistency was reported to be sufficient for all scales except for the descriptive scale of the rule orientation, which consists of three items (Cronbach’s α = .58), and the evaluative scale of the innovation orientation, which consists of four items (Cronbach’s α = .69). Validity was assessed in the initial study by experts of the international FOCUS group [28]. In our sample, internal consistency was sufficient for all scales except for the descriptive scale of the rule orientation, which consists of three items (Cronbach’s α = .65) and the evaluative scale of the rule orientation, which consists of eight items (Cronbach’s α = .39). For this reason, these scales (descriptive and evaluative scale of the rule orientation) were not included in the analysis.

### Controlling variables

#### Knowledge of resident sexuality

The subsection of the Dutch version of the ASKAS [24] was also used to assess the knowledge of resident sexuality. This section consisted of 26 questions, including items such as “sexual activity in an aged person is often dangerous to their health.” A correct answer was granted a score of 1, a wrong answer a score of 2, and when respondents chose the “I don’t know” option, a score of 3 was given. Total scores were calculated, ranging from 26 (perfect score, most knowledge) to 78 (least knowledge), and used for analyses. Internal consistency was proven to be sufficient in the study of Mahieu et al. (2013; Cronbach’s α = .80) and for our sample as well (Cronbach’s α = .83).

#### Participant characteristics and characteristics of employees’ careers

Gender (male/female), age (in years), level of education (low vocational, average vocational, high vocational, and high (higher professional level and university)), tenure within care (in years), and current function (caregiver, therapist, other) were assessed. Second, participation in training in handling resident sexuality (sexual education: “none,” “one,” or “more than one”) was assessed. Finally, care organization (labeled as A through F) and the reported presence of policy concerning resident sexuality (yes/no) were included.

### Analyses

Before data collection, a power analyses was performed to find an appropriate sample size, using the program G*power [30]. To reach a power level of .95, using an alpha of .05 and an effect size of .15, a minimal sample size of 166 respondents was required. As mentioned before, we included all cases that completed at least 80% of the dependent variable scale. The final sample size was 187, in contrast to a possible sample size of 168, when all cases would be excluded in which one or more items on the dependent variable were not completed.

Descriptive analyses were performed to assess participant characteristics. Means and standard deviations were assessed for continuous measures, percentages for categorical measures. More descriptive statistics were estimated to assess preliminary differences in attitudes regarding residents’ sexuality between groups of employees. *T*-tests were used for two categories: policy regarding sexuality within an organization (yes/no). Analysis of variances (ANOVA) was used for more than two categories: level of education, employer, function, and sexual education. Differences among continuous independent variables (age, years of tenure) were assessed using linear regression analysis.

Hierarchical OLS regression analyses were conducted to examine the effects on and the addition to the variance explained in the dependent variable: attitude toward resident sexuality. Knowledge of resident sexuality was entered in Model 1. Because of the expected interrelation between knowledge and attitude, the control variables age, level of education, and years of tenure were included in Model 2 (gender was excluded because of the overrepresentation of women). Employment (employer, one of the six organizations that were included in the study, labeled as A through F), policy regarding resident sexuality, current function, and training were included in Model 3. Finally, in the last model, our main independent variables of interest, person-centered care and the eight variables measuring organizational culture, were entered (Model 4). Dummy variables were constructed for four variables with categorical measures: level of education (average vocational is the reference category), employer (Organization B is the reference category), function (caregivers is reference category), and training (none is reference category). Values of .1, .05, and .01 on α level were used to test for significance of the *p* values. The .1 of *p* level is considered marginally significant, owing to the large number of variables in the model.

## Results

### Descriptive results

Descriptive analyses (see Table 2 for means (*M*) and standard deviations (*SD*)) have demonstrated that differences in attitude toward resident sexuality were found between groups of employees based on levels of education *F* (3,178) = 11.36, *p* < .01. Employees with high levels of education reported more positive attitudes than other employees; function *F* (2,181) = 10.88, *p* < .01. Therapists reported more positive attitudes than direct caregivers and other employees. Finally, attitudes were significantly influenced by the presence or absence of policy *t* (165) = − 3.79, *p* < .01. Employees, who reported that policy regarding resident sexuality was not present, were found to have a more positive attitude than employees who did report that policy was present in their RCF organization. Descriptive results from analyses between care staff of different employers cannot be reported owing to inequality in group sizes. No single effects were found for age, years of tenure, and if employees received training concerning resident sexuality (sexual education).

**Table 2:** Significant differences in attitude scores between groups

		Mean attitude score (*SD*)^c^
Level of education^a^		
	Low vocational	69.7 (15.3)
	Average vocational	64.6 (15.3)
	High vocational	55.7 (10.9)
	High^d^	52.3 (14.4)
Current function^a^		
	Caregiver	63.4 (15.4)
	Therapist^d^	48.0 (11.6)
	Other	63.3 (15.7)
Policy^b^		
	Yes	65.9 (17.2)
	No	56.8 (13.5)

^a^Results found through ANOVA analyses

^b^Result found through a *t*-test

^c^Higher scores imply lower attitude toward sexuality

^d^Post Hoc Tukey HSD tests showed differences between groups

### Organizational factors

Complete results for all models of the effects on care staff attitudes toward resident sexuality are presented in Table 3. Model 1 shows that, indeed, knowledge has a positive effect on attitudes *(F* (1,182) 24.39, *p* < .01), and this effect remains in all subsequent models (yet changes slightly in magnitude). More knowledge of resident sexuality goes together with a positive attitude toward resident sexuality. Model 2 shows that care staff with a ‘high vocational level’ (beta = −.22, *p* < .05 in Model 4) are found to have more positive attitudes toward resident sexuality than the ‘average vocational level’, the reference category. At first, the highest educated caregivers were also found to enhance more negative attitudes but this result diminishes as more variables are included. We found no effects of age on caregiver attitudes and years of tenure. In Model 3, significantly more negative attitudes were found in care staff of Employers B and F than Employer D, the reference category. This effect, however, diminished in Model 4, in which the culture of the organization and person-centered-care are included in the model. The presence of policy regarding resident sexuality (beta = .25, *p* < .05 in Model 4) was significantly and positively associated with the attitudes of care staff in Models 3 and 4.

**Table 3:** Results from hierarchical OLS regression analyses

	Model 1		Model 2		Model 3		Model 4	
Predictors	SE	β	SE	β	SE	β	SE	β
	*F* (1,182) 24.39, R^2^(A. R^2^) 0.12 (0.11)							
Knowledge	.12	.34***	0.11	0.26***	0.11	0.33***	0.16	0.26**
			*F* (6,171) 8.62, R^2^(A. R^2^) 0.23 (0.20)					
Age			0.13	−0.11	.013	0.06	0.19	−0.05
Level of education								
Low vocational			3.33	0.12	3.60	0.02	4.62	−0.02
Average vocational (ref.)			–	–	–	–	–	–
High vocational			2.91	−0.21***	3.15	−0.24***	3.84	−0.22**
High			2.85	−0.26***	5.29	−0.23*	6.26	−0.20
Years of tenure			0.15	0.06	0.16	−0.08	0.21	−0.06
					*F* (16,143) 5.67, R^2^(A. R^2^) 0.39 (0.32)			
Employer								
A					3.55	0.11	4.13	0.13
B					3.16	0.22***	4.42	0.04
C					4.44	0.89	5.20	0.11
D (ref.)					–	–	–	–
E					3.62	0.14	4.63	0.17
F					4.45	0.18**	5.33	0.19
Policy					2.43	0.26***	3.09	0.25**
Function								
Caregivers (ref.)					–	–	–	–
HCP					5.65	−0.10	6.33	−0.10
Other					4.38	0.11	5.26	0.23*
Sexual education								
None (ref.)					–	–	–	–
one					3.62	−0.06	5.02	−0.05
> one					6.01	−0.06	7.69	−0.08
							*F* (23,95) 3.02, R^2^(A. R^2^) 0.42 (0.28)	
Person-centered care							0.27	−0.23**
Org. culture ^a^								
D. support							0.37	−0.22*
D. innovative							0.43	0.17
D. goal							0.45	−0.00
E. support							0.48	0.12
E. innovative							0.77	−0.01
E. goal							0.57	−0.22

*p < .1

**p < .05

***p < .01

^a^D. = descriptive perspective and corresponding variable, E. = evaluative perspective and corresponding variable

In the final model (Model 4) 44% of the variance in attitudes toward resident sexuality was explained by all of the variables together, *F* (25, 93) = 2.94, *p < .01.* In this final model, person-centered care and the eight variables on the culture of the organization explained an additional 5% of the variance in attitudes. Person-centered care was found to significantly predict differences in attitudes (beta = −.22, *p* < .05). Employees reporting to provide more person-centered care, report more positive attitudes with regard to resident sexuality. Of the six included variables measuring organizational culture, only the descriptive measurement of the support orientation was marginally significant in predicting differences in attitudes (beta = −.22, *p* < .1). Employees reporting more supportive behaviors, policies, and procedures reported more positive attitudes toward resident sexuality.

## Discussion

Person-centered care was found to have a significant effect on the attitudes of care staff towards the sexuality of residents with dementia. Employees of care organizations, who feel they provide more person-centered care, have more positive attitudes toward the sexuality of residents. The person-centered care paradigm advocates that residents are viewed in a holistic and empowering way [21, 22]. The effect uncovered by the study of providing person-centered care on care staff attitudes toward resident sexuality seems logical, because sexuality is certainly a basic human need. However, residents’ sexual needs were not explicitly mentioned in the literature on person-centered care. Moreover, we learned through previous literature that the sexual behavior of residents with dementia often causes feelings of discomfort and concern in care staff and, as a result, sexual needs are ignored or even perceived as problem behavior [4, 7, 13–15]. Enhancing person-centered care and so achieving a more personal relationship between resident and care staff, leads to more positive attitudes regarding resident sexuality and fewer feelings of discomfort and concern.

Regarding the culture of the organization, only the descriptive part of the support orientation, including observable supportive behavior, procedures, and policy in the organization, marginally affected care staff attitudes toward resident sexuality. Care staff that report their care organization to be observably more supportive had more positive attitudes toward the sexuality of residents with dementia. Our results are in line with the assumption made by Roach (2004), who noted the influence of the culture of an organization on care staff attitudes.

Results also demonstrated that the presence of policy had a significant impact on attitudes of care staff. When reporting the absence of a policy considering resident sexuality in their organization, they had a more positive attitude toward resident sexuality. This result is in contrast with previous research, in which the presence of policy was perceived as having a positive influence on care staff attitudes [9, 20]. This contradiction might be explained in that care staff, who reported more positive attitudes toward resident sexuality, set high standards for care in general and experienced the absence of or minimal policy or guidelines as insufficient. Moreover, the content of the present policy or guidelines was unknown. A restrictive content focused on safety and prevention of hazards, rather than a supportive tone, could have caused this effect. However, this result still raises questions and more research need to be performed to explain this counterintuitive effect.

Also contrary to previous research [16], our sample did not show an effect of age or years of tenure. Although the samples of this previous study seems mostly comparable with our sample (e.g. gender and function distribution), there might be a cultural difference between the Dutch and British populations. Moreover, in the sample of Bouman et al. (2007), the residential care staff and the staff caring for residents without dementia are included; in our sample, the staff provides care exclusively to residents with dementia.

Finally, we found that knowledge of the sexuality of residents influenced care staff attitudes. This variable was included as a control variable in this study, although the results confirm previous research [10] that the care staff’s knowledge of resident sexuality influences their attitude greatly. Although this was not a main target variable, implications for clinical practice can be derived from this result.

### Strengths and limitations

This study is characterized by several strengths and limitations. A first strength is that this study is, to our knowledge, the first attempt to assess the effect of organizational factors on care staff attitudes toward sexuality of residents with dementia. Although assumptions were made in previous research, an actual assessment has not yet been undertaken [9, 14, 20]. Moreover, in the final analyses, we did control for individual factors that were proven to have an effect on the attitudes of care staff toward resident sexuality in previous studies (except for religious adherence). Throughout this study, a wider view and understanding of attitudes of care staff toward the sexuality of residents with dementia is provided.

A second strength lies in the data collection. The completion of the questionnaires was planned after scheduled (team) meetings. Remaining participant questions could be answered before, during, and after completion. Most questions were related to textual and lingual ambiguities; none were related to completing the knowledge question. During some team meetings, discussion arose on the topic of resident sexuality. The topic raised thoughts and concerns among care staff, as they potentially reevaluated their knowledge, attitude, and skills in responding to the sexual needs of residents with dementia.

This study also has limitations. First, although validated instruments are used, person-centered care and organizational culture are considered difficult to operationalize. For example, the definitions of both constructs are subject to different definitions.

Second, the way data were collected yielded a limitation, because care staff might have felt peer pressure to participate. Although both the author and trained student assistant emphasized that participation was voluntary and withdrawal was possible at any time, the care staff still chose to participate.

Third, we encountered limitations with the questionnaires. First, the way measures are granted in the knowledge section of the ASKAS questionnaire prompted a discussion among the authors. An incorrect answer was granted a “higher” score than the “I don’t know” option, which seems counterintuitive. It means that respondents who admit to not knowing the answer are perceived as having less knowledge of resident sexuality than respondents who gave an incorrect answer. However, this questionnaire has been well studied and validated [24], and was used several times in this way. Therefore, we decided to use the questionnaire in the prescribed way. Second, the rule orientation, in both the descriptive and evaluative part of the FOCUS questionnaire, proved insufficient in internal consistency and, therefore, was not included in the OLS hierarchical analyses. Although this questionnaire is well studied [28], the number of items per scale (variable) ranged from three to eight, which is very small. Cronbach’s α coefficient tends to be sensitive to only a small number of items [23]. Moreover, the outcome measures were divided over eight variables, which complicated statistical analyses. It did, however, give us a detailed look into the culture of the organization, based on a broadly used model of competing values [29].

A fourth limitation lies in the study’s rather small scale. Only one region in the Netherlands was selected, which is a limitation with regard to generalization. Moreover, in this region of the Netherlands, most residents are Christian (Catholic or Protestant), which is why we did not include religion as an individual factor [10].

Finally, the gender distribution was a limitation, because women were overrepresented in the sample. A gender effect on the attitudes could not be explored owing to this distribution. However, this distribution is representative of the actual situation in clinical practice, and this was also the experience of researchers who conducted previous studies [4, 10].

### Implications for practice and future research

Next to known benefits [21, 22], the enhancement of person-centered care in dementia care can improve care staff attitudes toward many areas of life, including sexuality. Providing person-centered care will not only influence attitudes toward resident sexuality but, for example, might also decrease agitation in residents [31]. Moreover, improvement of a supportive culture of organization can contribute to more positive attitudes toward resident sexuality. Providing an open and supportive culture will encourage care staff to express their feelings of discomfort openly and initiate an open discussion on resident sexuality. This open discussion will probably improve the possibility for residents to express and experience sexuality in the way they want.

However, neither implication is straightforward in its practical implementation. Both imply a profound change on the organizational level [32], such as providing time and opportunity to care staff to invest in more personal relationships with the residents they care for and have team meetings in which care related topics can be discussed. This, of course is far more comprehensive than providing a training program on resident sexuality. It seems to be worth the effort, because greater improvements in the QoL for residents with dementia can be expected from providing both person-centered care and a supportive culture in the care organization than can be expected from providing just a training program.

Furthermore, greater knowledge of resident sexuality was specifically found to benefit care staff attitudes toward resident sexuality. The way to improve this knowledge needs more detailed consideration, because participation in education in resident sexuality did not significantly influence these attitudes in our study. In previous research, greater knowledge was found as a result of a training program [17].

To further close the lacuna in research, the assumed influence of the attitude toward resident sexuality on the actual behavior of care staff needs further investigation. The behavior of care staff was found to be important, because they influence the possibility of residents and their possible partners expressing sexuality as they want. Spouses of residents mentioned, in qualitative research, that the behavior of care staff (both direct and indirect care staff) was important to their experiences of intimacy and sexuality [33]. However, a clear confirmation of the influence of care staff attitudes on their actual behavior concerning resident sexuality is lacking.

Finally, replication of this study, in another cultural setting, could add detail to the image that is presented here, especially as future generations of the elderly enter RCFs, and will probably demand more facilitation with regard to their intimate and sexual needs [34]. Moreover, the counterintuitive effect of the presence of policy on the attitude of care staff, needs further research.

## Conclusions

To establish a broader understanding of the attitudes of the care staff toward the sexuality of residents with dementia, it is important to know more about the organizational factors (person-centered care and the culture of the organization) that might influence factors these attitudes, next to factors on an individual level (e.g., age, level of education, and knowledge of resident sexuality). The aim of this study was to determine the effect of these two organizational factors.

Person-centered care was found to influence the attitudes and a supportive culture of the care organization was found to marginally influence care staff attitudes toward the sexuality of residents with dementia that they care for positively.

Moreover, knowledge of the sexuality of residents influenced care staff attitudes positively and the known presence of policy regarding resident sexuality influenced the attitudes negatively, which contradicted results from previous research.

## Abbreviations

QoL: Quality of life
RCF: Residential care facilities
AKSAS: The aging sexual knowledge and attitudes scale
P-CAT: The person-centered care assessment tool
ANOVA: Analysis of variances
